# The revenue generated from clinical chemistry and hematology laboratory services as determined using activity-based costing (ABC) model

**DOI:** 10.1186/s12962-015-0047-7

**Published:** 2015-12-08

**Authors:** Kasaw Adane, Zenegnaw Abiy, Kassu Desta

**Affiliations:** Unit of Quality Assurance and Laboratory Management, School Biomedical and Laboratory Sciences, College of Medicine and Health Sciences, University of Gondar, PO Box 196, Gondar, Ethiopia; School of Graduate Studies, St. Mary’s University, P.O. Box-150244, Addis Ababa, Ethiopia; School of Allied Health Sciences, College of Health Sciences, Addis Ababa University, P.O. BOX-9087, Addis Ababa, Ethiopia

**Keywords:** Revenue, Cost, Activity-based costing, Laboratory service, TASTH

## Abstract

**Background:**

The rapid and continuous growth of health care cost aggravates the frequently low priority and less attention given in financing laboratory services. The poorest countries have the highest out-of-pocket spending as a percentage of income. Higher charges might provide a greater potential for revenue. If fees raise quality sufficiently, it can enhance usage. Therefore, estimating the revenue generated from laboratory services could help in capacity building and improved quality service provision.

**Methods:**

Panel study design was used to determine revenue generated from clinical chemistry and hematology services at Tikur Anbessa Specialized Teaching Hospital, Addis Ababa, Ethiopia. Activity-Based Costing (ABC) model was used to determine the true cost of tests performed from October 2011 to December 2011 in the hospital. The principle of Activity-based Costing is that activities consume resources and activities consumed by services which incur the costs and hence service takes the cost of resources. All resources with costs are aggregated with the established casual relationships. The process maps designed was restructured in consultation with the senior staffs working and/or supervising the laboratory and pretested checklists were used for observation. Moreover, office documents, receipts and service bills were used while collecting data. The amount of revenue collected from services was compared with the cost of each subsequent test and the profitability or return on investment (ROI) of services was calculated. Data were collected, entered, cleaned, and analyzed using Microsoft Excel 2007 software program and Statistical Software Package for Social Sciences version 19 (SPSS). Paired sample *t* test was used to compare the price and cost of each test. P-value less than 0.05 were considered as statistically significant.

**Result:**

A total of 25,654 specimens were analyzed during 3 months of regular working hours. The total numbers of clinical chemistry and hematology tests performed during the study period were 45,959 (66.1 %) and 23,570 (33.9 %), respectively. Only 274, 386 (25.3 %) Ethiopian Birr (ETB) was recovered from the total cost of 1,086,008.09 ETB incurred on clinical chemistry and hematology laboratory tests. The result showed that, about 133,821 (12.32 %) ETB was revenue not collected from out-of-pocket payments that was paid for the services as a result of under pricing. The result showed that 18 out of 20 laboratory tests were under priced. The cost burden related to free Anti Retro-viral Therapy (ART) services was 285,979.82 (26.3 %) ETB.

**Conclusion:**

The cost per test estimated was significantly different to the existing price. About 90 % of the tests were under priced. This information could warn the hospital to reconsider resetting prices of these tests profitability ration less than 1. The revenue collected could help to build capacity, upscale quality, and sustainable service delivery.

## Background

Health laboratories are integral and essential component of health system [1–3]. They provide vital support; facilitate the initiation and monitoring of appropriate clinical and public health interventions [1]. Laboratory results provide invaluable tools for making decisions and improving the wellbeing of the patients’ [3, 4]. Despite this central role laboratory services have, strengthening it until recently received little or inadequate attention in many developing countries [1, 3].

In addition to this, the continuing and rapid growth of health care costs strain budget [5]. The cost of health care is not the only barrier to health care access; there is a wide range of affordability, availability and acceptability barriers severely affecting the most poorer groups [6].

These poorest people have the highest out-of-pocket (OOP) spending as a percentage of their income [7]. Changing the financing mechanisms from OOP payment towards a greater reliance of prepayment mechanisms is necessary to address the full range of access barriers [6]. Payment presents barrier to health care service utilization and accessing the basic quality health care service utilization which is still denied to many poor people of the world [6, 8]. OOP payments are not equitable in chain and have the effect of excluding the poor from health care access as they cannot afford to pay for medical care and withdraw from treatment [9]. As the result of payments, poor households are likely to sink even further into poverty because of the adverse effects of illness on their earnings and general welfare [10].

Healthcare reform in the financing strategies include revising user fee and retaining the collected money at the public health facilities [11]. Higher charges might provide a greater potential for revenue [12]. User fee is an important source of revenue for public health care facilities [13]. This important resource enable the health care providers to render quality health care services, rationalizing, and designing rules for fee waivers [13]. Revenue empowers the facilities to provide quality service, increase patient satisfaction, and determines ones willingness to pay [14]. The more the people are willing to pay the better is the chance health care facilities to provide quality services [14]. If fees raise quality sufficiently, it can enhance usage [4].

Efficiency is possible through improving outcome and/or reducing cost. Cost factors contributing for the cost of services was used to redesign the process and estimate the amount of revenue that could be increased as the result of reduced cost of rendering services [15]. Cost information has paramount importance for providing services at reasonable price and estimate the amount of revenue collected from patients. Firms use accurate cost of services for determining profitability and make long-term strategic and day-to-day operating decisions [13]. Nowadays, with the growth and development of advancement and changes in technology and effects on costs are very significant [16]. Therefore; it is vital to estimate revenue that could be collected from laboratory services and evaluate the profitability for the particular services for thinking and planning of better quality service [8]. The revenue collected in the hospitals could serve for planning for better quality services that are provided at government hospital.

## Methods

Tikur Anbesa Specialized Teaching Hospital is found in the center of Addis Ababa, the capital city of Ethiopia. The hospital has an area of 123,000 m^2^ and its building rests on an area of 45,000 m^2^ of land. It was reported that its construction cost of the building with some advanced medical equipments 21,605,399.00 ETB at the time of its inauguration on 3rd November 1973. There are 1262 various rooms from the basement to the eighth floor of the building. It has been undertaking medical services, providing training in the medical profession at different levels and conducting research activities in the fields of medicine and other sciences since 1974 [17]. The hospital is the highest referral hospital of the country and has been doing researches, teaching various disciples of students, and providing various degrees of specialized community services since its inauguration.

Panel study was designed to estimate the cost of rendering diagnostic laboratory testing services and to calculate the actual revenues collected from clinical chemistry and hematology laboratory services. In addition to this, the payment status of each request with all the service complex or compositions were observed along with the types of tests performed while grabbing data on all resources consumed during the process of diagnosis. The amount of revenue collected per test from service charge was estimated for each test. The laboratory test service charge or price was used for estimating the revenue generated from each tests and patient’s payment status was used while collecting data for revenue estimation. The service prices were fixed for each test. The service revenue collected from each laboratory test was estimated from the service bills, and/or patient receipts. ABC tool was used to assess the correct cost of services.

All resources consumed with activities were traced and/or allocated for costing laboratory services. All clinical chemistry and hematology laboratory examination sub processes or process maps were designed. While designing or analysis and designing of process maps work breakdown structures which could trap all resource inputs, the authors used direct observation using pre-tested data collection checklists, instrument user manuals, reagent insert kits, laboratory bench aids, and standard operating procedures.

Steps in estimating laboratory service revenue and profitability.

- *First:* identify procedures of interest (tests of clinical chemistry and hematology).
- *Second:* determine work breakdown structure or process maps.
- *Third:* determine all the resources associated to the testing processes.
- *Fourth:* establish cause/effect relationships with resources, activities and services.
- *Six:* collect the cost of services from activities and aggregate to the cost object.
- *Seven:* calculate the revenue from the services.
- *Eight:* compare the revenue and cost and determine the profitability of each test.

The historical cost and/or market prices of resources were collected form service bills, cost information or receipts. Data was collected from different department like purchasing, finance, property, marketing, clinical departments and other support departments. The cost and/or price information was collected from different departments of the hospital be it either from cost centers or revenue centers. We used flexible method of data collection. Payroll, service bills, models, office documents, agreement contracts were used both from the hospital and outside the hospital stakeholders supplying different reagents and materials as fixed asset or inventories. The service bills of water, electricity, telephone, and others were used as data. The depreciation cost of fixed assets was calculated using single line depreciation method. The cost of resources were assigned or allocated to the service based on the amount of services provided or resources consumed to the laboratory activities.

Resources used at various stages of the activities were observed and coded for easier data collection, entry and analysis. The cost of each of the resources was associated based on the amount of service or the cost of the resources consumed according to the relationships established by using the innovative costing model. Data were entered into Microsoft (MS) Excel 2007 spreadsheet program and analysis was performed using MS Excel 2007 spread sheet and SPSS (version 19.0, Chicago, IL, USA) programs. Cost versus price was compared using paired sample t-test. P ≤ 0.05 was used as a cut-off for considering statistical significance of the tests.

The study was conducted after formal approval letter obtained from the Department Research Ethics and Review Committee (DRERC) of the department of Medical Laboratory Sciences, School of Allied Health Sciences, College of Health Sciences, Addis Ababa University. An official letter was communicated to Tikur Anbesa Specialized Teaching Hospital. Institutional based formal approved letters were obtained from the hospital and then communicated to all conceivable departments.

## Result and discussion

The health care financing reform of Ethiopia states about the retention, utilization of revenues, and administration of fee waiver system and establishment of facility governance bodies. According to 2008/2009 performance report, there were 73 hospitals and 823 health centers have already started retaining revenues [18]. The strategies of the reform include revising user fees charged at government health care facilities. Price revision requires accurate cost information. The detailed research estimated correct cost information on which cost has to rely on. Basing on the cost information, citizens in government health facilities payment mechanisms were devised. These mechanisms depend on the type of services, whether the consumer have waiver certificate for non-exempted services. According to Healthcare Financing Reform Implementation Manual of 2010, government health facilities had been collecting very little revenue from user fees in comparison to the allocated budget from government treasury [11]. The revenue collected at hospital could serve to provide additional capacity to fulfill resources and hence to up scale the quality of services rendered to the patients. TASH could use this opportunity to improve the quality of service by building internal staff capacity through training providing resources.

During the study period a total of 25,654 specimens were collected and received for analysis. The total numbers of clinical chemistry and hematology tests performed during the study period were 45,959 (66.1 %) and 23,570 (33.9 %), respectively. Among these 15,028 (58.6 %) of them were hematology specimens and the remaining were clinical chemistry specimens. Among all specimens sent for analysis, 12,793 (49.87 %), 12,636 (49.26 %) and 228 (0.88 %) of specimens were with the payment status of free, paid and with unknown payment status, respectively. This indicates that nearly half of the total specimens are free laboratory service beneficiaries. From which clinical chemistry specimens account about 5556 (52 %), 4842 (46 %) and 228 (2 %) of free, paid and unknown payment status, respectively. Similarly, from 7794 (52 %) hematology specimens revenue was collected but from remaining not. This differs to the findings of the research done in Gondar from secondary data where 46,178 (17.7 %) of the patients were free health service beneficiaries. The reason for difference could be due to the difference in methodology, time, socio-economic status [19]. On the other hand, the result of facility exit survey conducted in Jimma, southwest reported about 58 % of the respondents were free health care beneficiaries [20]. The gap among these three studies may be due to the absence of clearly stated criteria for recruiting and providing waive certificate as it is suggest by the two previous studies [19, 20] and failure to serve the disadvantaged and vulnerable groups. There might be missing of the vulnerable and including those who do not deserve. However; currently criteria is set to which group of society waiver certificate is to be given. The general problem regarding the implementation of free certificate provision for the needy might be the case for the differences among studies. The problem might continue on the practice of providing to the real poor who deserves free services at public health facilities. The screening systems justifying the real evidence of poverty should be balanced and well addressed and those who provide waived certificate should take the responsibility on behalf of free health care beneficiaries.

Financial protection from all forms of costs of healthcare and enabling access to all citizens are key elements for universal health coverage. However, relatively limited progress towards universal coverage has been made in African countries [21]. The experience of Guinea, Nigeria and Cameroon showed that 70 % or more was financed through out-of pocket payment which is more than the result of our study. On the contrary, it is more than the experience of Ghana and Rwanda ensured universal health coverage and sustainable financial protection through domestic pre-payment financing [21]. The difference that occurred among these countries may be due to the socioeconomic differences, study setting and differences in the government policy differences. It may also be due to the setting where the research was done. The experience of the mentioned Africa countries is gross and our report is research output done in one referral hospital where the service mix is quite different from the overall picture of the country wide health system. Our countries perhaps use either one or a combination of both financing mechanisms and still other better mechanisms may be devised. The health care financing mechanism(s) should be implemented as far as it does not affect the health care seeking behavior of the citizens. These might be due to financing mechanisms and the socioeconomic difference of the countries. It differs to a study conducted in Indian in 2010 where only 10 % of patients got free laboratory and diagnostic services. Another 50 % were offered subsidized rates or a lower affordable price while remaining 40 % were charged in the private category [22]. The difference might be due to policy difference and socioeconomic of two countries. The hospital could use the experience of India to collect more revenue after requesting the change in the policy issues.

The total cost of clinical chemistry and hematology laboratory services was about 1,008.09 ETB. From this amount of health care the hospital expended, only 274,386 (25.3 %) ETB was collected from patients during the study period. This revenue is lower than 37 % of money which is managed by household out-of-pocket payments as of the 2007/08 National Health Expenditure [21]. This may be due to under pricing. The total cost of tests from HIV positive individuals was 285,979.82 (26.3 %) ETB. All of them were exempted for laboratory test services. According to the result of our study 140,849 (37.1 %) ETB was collected from the total known cost of 380,963.63 (97.9 %) ETB paid and free served clinical chemistry tests is depicted in (Table 1) and 7934 (2.1 %) ETB costs of the tests were with unknown payment status which is not displayed in (Table 1). This is the main reason for the very low amount of revenue collected from OOP payments is due low 0.88 average profitability ratio (PR) or return on investments (ROI). In addition to this service were under priced (see Table 1) for PR or ROI or price. The revenue from clinical chemistry paid services was less with an amount 19,846.41 (5.2 %) ETB. From a total cost of Br. 648,935.8 (100.00 %) for hematology tests only 133,537 (19.16 %) ETB revenue was collected through user fee OOP payments. This was less than 113,974.20 (17.6 %) ETB that could have been collected with PR equal to 1 which is in fact less than an ideal cost for non profitable public service price setting, though there is a need to mark up price is necessary. The internal comparison for clinical chemistry and hematology showed better performance for clinical chemistry service profitability. This amount of money is part of the hospital unused capacity. Relatively lower gross amount of revenue loss from clinical chemistry tests might be due to marked up pricing of LDL-C and TG tests. Had price been set on the cost of services 37.6 % of the service cost would have been recollected. This would have been similar to 2007/08 NHE 37 % which was reported to managed and financed through OOP payment [21].

**Table 1:** The total cost, and profitability to prices charged on tests at TASH, Addis Ababa, Ethiopia, October–December 2011

Test profile	Free		Paid		Total		PR
	Cost	Price	Price	Cost	Cost	Price	
Cr	31,956	28,315	29,555	26,188	62,861	55,700	0.89
Urea	33,136	28,287	30,644	26,160	65,191	55,651	0.85
AST	25,582	22,218	22,384	19,440	49,046	42,596	0.87
ALT	25,502	22,176	22,308	19,398	48,889	42,512	0.87
ALP	25,229	21,938	22,034	19,160	48,349	42,043	0.87
CHO	11,660	9905	4029	3423	15,977	13,573	0.85
FBS	22,917	20,027	11,615	10,150	35,060	30,639	0.87
LDL	4069	6100	1641	2460	5776	8660	1.5
Tg	10,868	20,175	3701	6870	14,835	27,540	1.86
HDL-C	25,978	13,370	8880	4570	35,499	18,270	0.51
STP	3111	2424	3604	2808	6853	5340	0.78
CSFP	262	192	303	222	573	420	0.73
Clin. che. total	*220170*	*195127*	*160698*	*140849*	*388909*	*342944*	*0.88*
CBC	192967	104705	196411	106483	389378	211188	0.54
PCV	4539	1576	9919	3444	14458	5020	0.35
ESR	12134	7030	15586	9030	27720	16060	0.58
ABO	10302	6060	20135	11844	30437	17904	0.59
BM	1454	700	2416	1155	3870	1855	0.48
Malaria	1747	845	1634	790	3381	1635	0.48
CD4 +	177282	0	0	0	177282	0	0
RBS	999	560	1411	791	2411	1351	0.56
Hemat total	*401425*	*121476*	*247511*	*133537*	*648936*	*255013*	*0.39*

*ABO* blood group; *BM* blood morphology; *CBC* complete blood count; *CHO* cholesterol; *CI* confidence interval; *Cr* creatinine; *CD4+* CD4+ test; *ESR* erythrocyte sedimentation rate; *FBS* fasting blood sugar; *PCV* packed cell volume; *df* degree of differentiation; *AST* aspartate transaminase; *ALT* alaninine transaminase; *ALP* alkaline phosphatase; *TG* triglyceride; *HDL-C* high density lipoprotein cholesterol; *LDL* low density lipoprotein; *RBS* random blood sugar; *STP* serum total protein; *CSFP* cerebrospinal fluid total protein; *Hemat* hematology

The result of our study showed that the profitability ratio (PR) of 18 from 20 tests did not reach 1. On the other hand, in Thai study only 6 out of 53 did not reach PR 1. The average PR of our study was 0.75 while in Thai study it was 4.46. The actual PR in our study was 0.26 in contrasts to this it was 3.3 in Thai study, which was calculated when the actual revenue divided to the actual cost was. PR was 0.88 for clinical chemistry and 0.39 for hematology tests (Table 1).

The possible reason for intra study and the inter study PR difference may be due to the policy and the pricing inefficiency [23]. There was a statistically significant difference between cost and price for all tests with P-value <0.001 for all tests (Table 2). Only two from twenty tests were priced with some mark up but the remaining test profiles were under priced. The numbers of tests performed per day were significantly different with (P < 0.001). This may be due to the schedule of the hospital’s departments and stock out of reagent and machine down time.

**Table 2:** Cost versus price difference of tests comparison of clinical chemistry and hematology tests performed at TASH, Addis Ababa, Ethiopia, Oct. 2011 to Dec. 2011

Paired samples test						
	Paired differences					Sig.(2-tailed)
	Mean (95 % CI of difference)	SD	SE of mean	t	df	p-value
Clinical chemistry						
Cr	0.674 (0.667–0.068)	0.39	0.004	177.952	10625	0.000
Urea	0.898 (0.888–0.908)	0.521	0.005	177.641	10625	0.000
AST	0.607 (0.597–0.617)	0.524	0.005	119.314	10625	0.000
ALT	0.600 (0.590–0.610)	0.520	0.005	119.039	10625	0.000
ALP	0.593 (0.584–0.603)	0.521	0.005	117.519	10625	0.000
CHO	0.226 (0.217–0.235)	0.479	0.005	48.698	10625	0.000
FBS	0.416 (0.407–0.425)	0.497	0.005	86.268	10625	0.000
LDL	−0.271 (−0.289 to −0.254)	0.911	0.009	−30.704	10625	0.000
Tg	−1.196 (−1.245 to −1.246)	2.616	0.025	−47.109	10625	0.000
HDL-C	1.625 (1.554–1.689)	3.558	0.035	46.970	10625	0.000
STP	0.142 (0.133–0.151)	0.471	0.005	31.165	10625	0.000
CSFP	0.014 (0.011–0.017)	0.171	0.002	8.394	10625	0.000
Hematology						
CBC	11.85 (11.78–12.92)	3.76	0.03	393.569	15027	.000
PCV	0.63 (0.59–0.66)	2.08	0.02	37.003	15027	.000
ESR	0.78 (0.75–0.80)	1.49	0.01	63.911	15027	.000
ABO	0.83 (0.81–0.86)	1.68	0.01	61.014	15027	.000
Malaria	0.12 (0.10–0.13)	0.78	0.01	18.283	15027	.000
CD4+	11.79 (11.22–12.37)	35.82	0.29	40.364	15027	.000
BM	0.13 (0.12–0.15)	0.85	0.01	19.404	15027	.000
RBS	0.07 (0.06–0.08)	0.62	0.01	13.982	15027	.000

*ABO* blood group; *BM* blood morphology; *CBC* complete blood count; *CHO* cholesterol; *CI* confidence interval; *Cr* creatinine; *CD4+* CD4+ test; *ESR* erythrocyte sedimentation rate; *FBS* fasting blood sugar; *PCV* packed cell volume; *df* degree of differentiation; *AST* aspartate transaminase; *ALT* alaninine transaminase; *ALP* alkaline phosphatase; *TG* triglyceride; *HDL-C* high density lipoprotein cholesterol; *LDL* low density lipoprotein; *RBS* random blood sugar; *STP* serum total protein; *CSFP* cerebrospinal fluid total protein; *Hemat* hematology

It is much great surprising to report or say 18 out of 20 services were under priced and did not reach with profitability ratio 1. The same could happen to any other services delivered to the patients by TASH and even other health institutions of the country. The government could give emphasis to plan such huge capacity that could help to scale up the quality of services. In general there is big gap between the cost and the price set for services rendered in exchange for laboratory services. The hospital should give much attention to costing even any other services provided in the hospital. The revenue generated could help to hospital to plan for more improved quality services by using unused capacity which is an important resource or good opportunity for hospitals to think about better quality services at all settings.

## Conclusion

The total revenue collected from services provided during the study period was 274,386 (25.3 %) ETB. The total cost of services of clinical chemistry and hematology was 1,086,008.09 ETB. About 133,821 (12.32 %) ETB was not collected as a result of under pricing (Table 1). The ART service burden was about 285,979.82 (26.3 %) ETB. The cost per test was significantly different from the price charge for all laboratory services provided in the hospital. Only two tests from 20 were priced with some mark up, but the remaining 18 test profiles were under priced and the average profitability of clinical chemistry was 0.88 and that of hematology tests was 0.39. The overall revenue collected from clinical chemistry and hematology laboratory services was about a quarter of the total cost incurred for rendering service during the study period. The government could use this result so as to think of more researches and take timely action so as to revise user fees at public health institutions and use the money generated for planning more improved service provision. Nearly half of the patients are free certificate user. Therefore; the hospital, the government and any stakeholders involved in providing waived certificate could take time to think and revise user certificate provision systems and make it more clear, transparent, economical to identify those who deserve and enable them access the certificate effectively so as to increase the universal health care service coverage. The revenue could help the hospital to build up the capacity for more efficient, improved quality health care services. ABC tool could be used for correct cost estimation, revenue estimation of profitability analysis, efficient resource allocation, and improved quality service provision. There is a great gap between and the cost of laboratory services and price set for them. Hence, the hospital should adapt better costing methods rather than going simply with traditional methods of costing. For this the authors would like to suggest the best costing model ABC while implementing costing strategies.

## Strength and limitation of the study

The strength of our study was a detailed cost analysis of the laboratory services using ABC and large sample size. The limitation of this study is that the involvement of internship students in the activities the diagnostic services was not considered. We did not also consider the fixed assets used for more than useful life years.

## Abbreviations

ART: anti-retroviral therapy
ABC: activity based costing
ALP: alkaline phosphatase
ALT: alanine transaminase
AST: aspartate aminotransaminase
BM: blood morphology
TB: Ethiopian Birr
CBC: complete blood count
CHO: cholesterol
Cr: creatinine
ESR: erythrocyte sedimentation rate
FBS: fasting blood sugar
HDL-C: high density lipoprotein cholesterol
HIV: human immuno-deficiency virus
OOP: out-of-pocket
LDL-C: low density lipoprotein cholesterol
NHE: National Health Expenditure
ROI: return on investment
RBS: random blood sugar
PCV: packed cell volume
PR: profitability ratio
SP: serum protein
SPSS: statistical software package for social sciences
TASTH: Tikur Anbessa specialized Teaching Hospital
TG: triglyceride
